# Fruit and Vegetable Consumption and Its Public Health Implications for Hypertension Among Peruvian Aged 15 Years and Older: Evidence from the 2022–2023 Demographic and Health Survey

**DOI:** 10.3390/healthcare14050566

**Published:** 2026-02-25

**Authors:** Miguel Ipanaqué-Zapata, J. Franco Rodriguez-Alarcon, Joel Figueroa-Quiñones, Hessel Valle-Sandoval, Oriana Rivera-Lozada, Paolo Cayetano-Terrel, Janina Bazalar-Palacios, Alberto Cerna-Salcedo

**Affiliations:** 1 Vicerrectorado de Investigación, Universidad Señor de Sipán, Chiclayo 14001, Peru; paolocayetanoterrel@gmail.com (P.C.-T.); csalcedoalbe@uss.edu.pe (A.C.-S.); 2 Facultad de Medicina Humana, Universidad Ricardo Palma, Lima 15039, Peru; franco.investigacionu.peru@gmail.com; 3 Escuela de Psicologia, Universidad Autónoma de Ica, Ica 11701, Peru; joelfq.13@gmail.com; 4 Facultad de Ingeniería y Arquitectura, Universidad Católica de Trujillo, Trujillo 13600, Peru; hessel.vs5@gmail.com; 5 Facultad de Ciencias de la Salud, Universidad Tecnológica del Perú, Lima 15046, Peru; jbazalar@utp.edu.pe

**Keywords:** hypertension, fruit and vegetable consumption, Peru, Demographic and Health Survey, obesity, cross-sectional study

## Abstract

**Background:** Hypertension (HTN) remains a major non-communicable disease globally and a leading cause of cardiovascular morbidity. Fruit and vegetable consumption (FVC) has been widely promoted as a preventive strategy, yet post-pandemic evidence from low- and middle-income countries remains limited. **Objective:** This study aimed to examine the association between the consumption of five or more servings of fruits and vegetables and hypertension among the Peruvian population during the post-COVID-19 period. **Methods:** A cross-sectional analytical study was conducted using data from the 2022–2023 Peru Demographic and Health Survey (DHS). The final weighted sample included 64,347 individuals aged 15 years and older. Multinomial logistic regression and robust Poisson regression models were applied, accounting for complex survey design and sampling weights, to estimate relative risk ratios (RRR), prevalence ratios (PR) and 95% confidence intervals (CIs) for pre-hypertension, hypertension and its subtypes. **Results:** Overall, 14.20% of the Peruvian adult population presented with hypertension. Consumption of fruits and vegetables was not significantly associated with either pre-hypertension or hypertension (*p* > 0.05). In contrast, older age (aRRR = 47.55; *p* < 0.001) and obesity (aRRR = 7.16; *p* < 0.001) were strongly associated with hypertension. When classifying hypertension by subtype (systolic or diastolic), fruit and vegetable intake remained non-significant (*p* > 0.05), while obesity exerted a stronger effect on diastolic hypertension (aPR = 9.04; *p* < 0.001). **Conclusions:** No significant association was found between fruit and vegetable consumption and hypertension among Peruvians during the post-COVID-19 period. However, age and body mass index were key determinants of both pre-hypertension and HTN, highlighting the need for broader, integrated public health strategies beyond dietary promotion alone.

## 1. Introduction

Hypertension (HTN) is a major global public health challenge. It affects approximately 1.28 billion adults and contributes substantially to premature mortality through its close relationship with diabetes, cardiovascular disease, and obesity [1,2]. Although prevalence varies by region—with comparatively lower levels in the Americas [1]—the Peruvian context remains concerning. National reports and recent reviews indicate a substantial and heterogeneous HTN burden across subpopulations and geographical areas, with important clinical and economic implications for the health system [3,4]. Hypertension, in addition to its high prevalence, causes damage to target organs (heart, kidneys, retina, and brain), lowers quality of life, and raises the risk of cognitive impairment, cardiovascular events, and death [5,6,7,8].

During the post-COVID-19 period, although many daily activities gradually returned to pre-pandemic patterns, persistent disruptions in access to fresh foods and sustained changes in weight- and physical-activity-related behaviours were observed, particularly among specific population groups and geographic areas. These residual effects may have implications for blood pressure control [9,10]. In this context, estimating the independent association between fruit and vegetable consumption (FVC) and HTN requires rigorous adjustment for key demographic, socioeconomic, and anthropometric determinants—especially age and body mass index (BMI)—and careful consideration of the potential for residual confounding inherent to population-based survey data [11,12,13,14,15].

The scientific evidence regarding the association between FVC and HTN remains inconsistent. This variability may be partly explained by differences in how FVC is operationalised (e.g., frequency, portion size, grams/day, total intake, or intake quantiles). Studies using more detailed exposure metrics often report an inverse association, whereas analyses relying solely on the “five-a-day” threshold—particularly those assessing independent effects after adjustment for age, BMI, and other health-related behaviours—frequently show attenuation or null findings [16,17,18,19]. Despite the relevance of broader dietary patterns (e.g., sodium intake, dietary diversity, or the Dietary Approaches to Stop Hypertension (DASH) diet), FVC remains a distinct and policy-relevant dietary indicator. It is widely used in global surveillance systems, including the DHS, due to its simplicity, cross-country comparability, and alignment with WHO public health recommendations. Moreover, FVC may capture specific biological mechanisms—such as higher potassium and fibre intake and vascular benefits—that may operate partly independently of overall dietary patterns [20]. Biologically, higher FVC intake may contribute to blood pressure reduction through increased potassium and fibre intake and favourable effects on vascular function [14].

Previous studies have consistently shown that hypertension is shaped by a combination of demographic, socioeconomic, and anthropometric factors. Age and BMI are among the strongest determinants of elevated blood pressure, while sex differences have also been widely documented across populations [21,22,23,24]. In addition, socioeconomic position, marital status, and place of residence have been associated with HTN risk through behavioural, environmental, and contextual pathways in population-based studies [25,26,27,28]. These factors were therefore included as covariates to minimise confounding and ensure comparability with existing epidemiological evidence.

Beyond blood pressure, FVC may also be relevant to broader aspects of cardiovascular regulation, including resting pulse rate. Diets rich in fruits and vegetables provide potassium, dietary fibre, antioxidants, and other bioactive compounds that may favourably influence vascular tone, endothelial function, and autonomic balance—physiological mechanisms closely linked to both blood pressure regulation and heart rate control [14,18,23]. From a public health perspective, these shared pathways suggest that fruit and vegetable intake may have cardiovascular implications beyond blood pressure outcomes alone, even when independent associations with hypertension are not consistently observed.

Across the existing evidence, findings on the association between FVC and HTN remain heterogeneous. In Ghana, among 4168 women aged 15–49 years, adherence to the “five servings per day” recommendation was not independently associated with hypertension after adjustment for age and BMI [26]. Similarly, in Bangladesh, a study of 6094 adults aged ≥25 years found no significant association between FVC—modelled as a continuous variable—and hypertension [29]. In contrast, evidence from Ethiopia indicates a different pattern: among 518 urban residents, low vegetable consumption (≤3 days/week) was associated with a higher prevalence of HTN, alongside elevated associations for physical inactivity and added salt intake [21]. A population-based study in Iran involving 29,378 participants reported a significant inverse relationship with fruit consumption (>2 portions/day), whereas no association was found with vegetable intake [17]. Finally, in China, among 18,757 adolescents aged 13–17 years, consumption of ≥3 servings of fruits and vegetables per day was associated with a lower risk of HTN [22]. These mixed findings highlight the context-dependent nature of the FVC–HTN relationship and the need for population-specific analyses using standardised exposure definitions.

Against this backdrop, the present study asked whether FVC is associated with hypertension in the 2022–2023 period. Specifically, our objective was to evaluate the association between FVC and HTN among Peruvian aged 15 years or older, explore the relationship with hypertension subtypes (isolated systolic, isolated diastolic, and combined), and estimate their prevalence in the study population.

The knowledge gap addressed here stems from inconsistencies in the international literature regarding the FVC–HTN relationship and from the scarcity of recent studies in Peru, particularly during the post-confinement period following COVID-19. Our aim was to generate evidence relevant to the prevention and control of hypertension within national public health and clinical practice.

## 2. Materials and Methods

### 2.1. Study Design

This cross-sectional study involved a secondary data analysis of the Peruvian Demographic and Health Survey (DHS, known locally as ENDES) for the years 2022–2023. The DHS is a nationally representative, population-based survey designed to collect comprehensive public health data covering both communicable and non-communicable diseases [23].

The DHS-Peru employs a complex, two-stage, stratified, probabilistic sampling design at the departmental level, differentiated by urban and rural areas, to ensure population-level generalization [23]. The survey is implemented by the National Institute of Statistics and Informatics (INEI), and the corresponding datasets are publicly available at: https://proyectos.inei.gob.pe/microdatos/ (accessed on 31 November 2025).

### 2.2. Participants

The study population included individuals aged 15 years and older residing in Peru. The DHS-Peru individual samples were validated annually, with unweighted sample sizes of 31,352 (2022) and 31,445 (2023). Weighting factors were applied to ensure population-level estimates, resulting in weighted sample sizes of 35,824 (2022) and 35,814 (2023).

**Figure 1:**
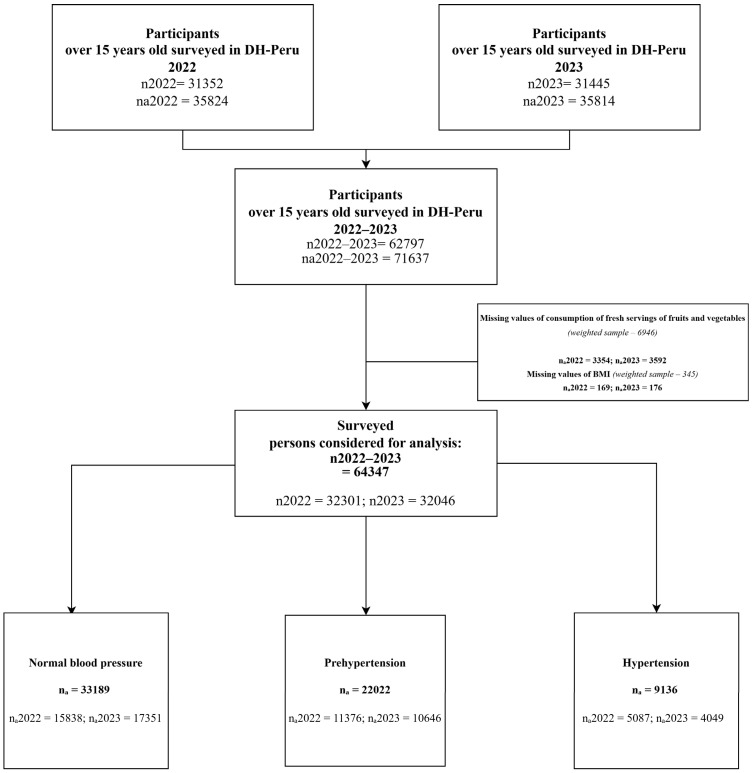
**Flowchart of study participants. n_2022_** and **n_2023_** = unweighted sample size; **n_a_**, **n_a2022_** and **n_a2023_** = weighted sample size. For the present analysis, the weighted sample was used.

Participants with missing information on fruit and vegetable consumption (*n* = 6846) or body mass index (*n* = 345) were excluded. The final analytical sample consisted of 64,347 participants (Figure 1).

### 2.3. Variables

#### 2.3.1. Outcome Variable

The outcome variable was HTN, defined as elevated blood pressure [25]. For each participant, blood pressure was taken twice, including systolic blood pressure (SBP) and diastolic blood pressure (DBP). Measurements were obtained with the participant in a seated position, with the first reading taken after five minutes of rest and a second reading obtained two minutes later [25,26].

SBP was obtained from questionnaire items QS903S (first measurement) and QS905S (second measurement), while DBP was obtained from QS903D (first measurement) and QS905D (second measurement). The average of both measurements was used to calculate the final SBP and DBP values. However, when the absolute difference between the two SBP readings was ≥20 mmHg, only the second measurement was used. Similarly, if the absolute difference between DBP readings was ≥10 mmHg, only the second measurement was used. These procedures followed the indicator framework established by the INEI for multidimensional poverty assessment [26].

According to the Seventh Report of the Joint National Committee (JNC 7) on the prevention, detection, evaluation, and treatment of high blood pressure, blood pressure was classified into three categories: normal (SBP < 120 mmHg and DBP < 80 mmHg), pre-hypertension (SBP 120–139 mmHg or DBP 80–89 mmHg), and hypertension (SBP ≥ 140 mmHg or DBP ≥ 90 mmHg) [27]. Although more recent international guidelines recommend lower diagnostic thresholds, the use of JNC 7 criteria in this study ensures consistency with national surveillance data and facilitates comparability with prior population-based studies conducted in Peru and other low- and middle-income countries [30].

For analytical purposes, three binary variables were generated to classify specific hypertension subtypes: isolated systolic hypertension (ISH), isolated diastolic hypertension (IDH), and concurrent systolic–diastolic hypertension (CSDH). Each variable was coded as “1” if present and “0” otherwise. ISH was defined as SBP ≥ 140 mmHg and DBP < 90 mmHg; IDH as SBP < 140 mmHg and DBP ≥ 90 mmHg; and CSDH as SBP ≥ 140 mmHg and DBP ≥ 90 mmHg [19].

#### 2.3.2. Independent Variable

The main exposure variable was fruit and vegetable consumption, derived from two items in the DHS health questionnaire: daily fruit intake (QS213C) and daily vegetable intake (QS219U). Reported daily intake was converted to weekly consumption by multiplying each value by seven. Based on WHO recommendations for adequate intake (≥5 servings per day, equivalent to ≥35 servings per week), fruit and vegetable consumption was categorised into two groups: inadequate consumption (<35 servings per week) and adequate consumption (≥35 servings per week) [19].

#### 2.3.3. Other Variables

The covariates considered for the present study were sex, education level, age, marital status, wealth index, region, area of residence, body mass index (BMI), and years of education.

#### 2.3.4. Statistical Analysis

The analysis was conducted in Stata 16.1 (StataCorp, College Station, TX, USA), declaring the complex sampling design of the Peru DHS using svyset, incorporating the sampling weight (PESO15_AMAS), strata (HV022), and primary sampling units (PSU; HV001); the secondary sampling unit was optionally specified (SSU: household, HV002). Variables were characterised using univariable and bivariable analyses. Descriptive statistics are presented as weighted estimates with 95% CIs; between-group comparisons were assessed using the Rao–Scott χ^2^ test (*p* < 0.05). Subpopulation analyses were performed using the subpop () option.

To address the primary objective, the association between FVC and HTN categories (normal, pre-hypertension, and hypertension) was evaluated using multinomial logistic regression under the complex survey design, reporting relative risk ratios (RRR) and adjusted relative risk ratios (aRRR) with 95% CIs. Subsequently, the association of FVC with dichotomous hypertension phenotypes—ISH, IDH, and CSDH—was assessed using Poisson regression with robust variance and the complex design, reporting prevalence ratios (PR) and adjusted prevalence ratios (aPR) with 95% CIs.

For both models, unadjusted and adjusted estimates were reported. In the adjusted models, covariate inclusion was restricted to variables that showed a statistically significant association with the outcome in bivariable analyses (*p* < 0.05). This data-driven approach was adopted due to the absence, within the DHS, of several key covariates with established biological plausibility—such as detailed dietary factors and behavioural determinants—thereby limiting the ability to specify a causal multivariable model.

#### 2.3.5. Ethical Considerations

This study involved a secondary analysis of DHS data. The DHS was conducted with prior ethical approval, and informed consent was obtained from all participants. The datasets are anonymised and publicly available through the INEI data portal (https://proyectos.inei.gob.pe/microdatos/ accessed on 31 November 2025).

## 3. Results

The descriptive results and adjusted models are presented below, in accordance with the analytical plan (Table 1, Table 2 and Table 3; Figure 1). We first report weighted descriptive statistics (univariable) and bivariable comparisons across blood pressure categories using the Rao–Scott χ^2^ test (α = 0.05) (Table 1). In the weighted sample (*n* = 64,347), 14.20% had hypertension and 34.22% had pre-hypertension. Approximately four in ten men had pre-hypertension (44.33%). The prevalence of elevated blood pressure increased with age, with markedly higher values than in the 15–19-year group (difference = 31.16). With respect to BMI, a higher proportion of hypertension was observed among individuals with obesity (18.29%). In bivariable analyses, FVC and the covariates differed significantly across blood pressure categories (Rao–Scott χ^2^, *p* < 0.05).

Regarding factors associated with pre-hypertension and HTN, all variables were statistically significant in the crude analyses (*p* < 0.05). However, in the adjusted model, FVC was not independently associated with either pre-hypertension or HTN (*p* = 0.17 and *p* = 0.33, respectively). Sex, age, marital status, wealth index, region, area of residence, and body mass index (BMI) were associated with both outcomes (*p* < 0.05), except area of residence in relation to HTN (*p* = 0.11). Adults aged ≥ 60 years had a substantially higher prevalence of pre-hypertension and HTN (aRPR = 5.25 and aRPR = 47.55, respectively). In addition, individuals in higher wealth categories showed a higher prevalence of pre-hypertension (aRPR = 1.81) and HTN (aRPR = 1.35). Finally, obesity was strongly associated with both pre-hypertension (aRPR = 4.22) and HTN (aRPR = 7.16) (Table 2).

We examined HTN subtypes—isolated systolic (ISH), isolated diastolic (IDH), and combined systolic–diastolic (CSDH)—using Poisson regression with robust variance, reporting crude and adjusted models. In crude analyses, FVC was associated with IDH and CSDH, with lower consumption associated with a higher prevalence (*p* < 0.05), whereas the association with ISH was not statistically significant (*p* = 0.51). In adjusted models, FVC was not independently associated with any subtype (*p* > 0.05). By contrast, obesity showed strong independent associations with IDH and CSDH (aPR = 9.04 and aPR = 9.43, respectively; *p* < 0.001), with additional gradients by age and other covariates (Table 3).

## 4. Discussion

In the main analyses, FVC was not independently associated with either pre-hypertension or HTN after multivariable adjustment. This aligns with reports in which the apparent protective signal of fruit and vegetable intake, or plant-forward dietary patterns, attenuates after controlling for lifestyle and dietary covariates [17,26,30]. Among Chinese adolescents, higher vegetable intake (>2 portions/day) was associated with lower odds of hypertension (OR = 0.66; *p* < 0.05), suggesting a protective association rather than an increased risk [22]. Taken together, these mixed findings indicate that crude associations between FVC and blood pressure are sensitive to how intake is measured and to the extent of confounder control.

Mechanistically, higher fruit and vegetable intake has been proposed to increase potassium intake, thereby promoting urinary sodium excretion and contributing to blood pressure reduction through modulation of the renin–angiotensin–aldosterone system and vascular tone [29]. In addition, potential pathways linking fruit and vegetable intake to blood pressure and adiposity include increased nitric oxide bioavailability via dietary nitrate [31], the actions of polyphenols/anthocyanins [32], and the physiological effects of dietary fibre [33]. By contrast, robust dietary frameworks such as the DASH pattern have consistently demonstrated benefits for blood pressure control and are endorsed for cardiometabolic risk management [34,35].

Likewise, evidence regarding the association between FVC and blood pressure outcomes in low- and middle-income countries has been inconsistent. A study by Yaya et al. (2018) in sub-Saharan Africa reported a protective association between fruit and vegetable consumption and the odds of hypertension, although it was also associated with higher odds of overweight/obesity [36]. In contrast, Mishra et al. (2005), in Uzbekistan, reported a counterintuitive association between fruit and vegetable intake and hypertension risk, alongside an increased risk of obesity [37]. Methodological differences may partly explain these discrepancies: Yaya et al. used a non-WHO-based measure to classify adequate versus inadequate vegetable consumption, whereas Mishra et al., similar to the present study, applied a WHO-based definition of adequate intake. Overall, in settings such as Peru, the relationship between FVC and blood pressure appears context-dependent, and further research is warranted to clarify the intake levels associated with clinically meaningful differences in hypertension and obesity, thereby informing future public health strategies.

Adults aged 60 years and over are at the highest risk of developing HTN or pre-hypertension. Consistent with this finding, the U.S. National Health and Nutrition Examination Survey (NHANES) reported that roughly 70% of adults aged > 65 years in the United States have hypertension [38]. Chen et al. [39] similarly observed that patients aged > 60 years often display complex blood pressure patterns that are more difficult to control; however, they emphasised that intensive management in this age group can prevent serious cardiovascular consequences. This heightened predisposition to elevated blood pressure among older adults is largely attributed to vascular ageing [40]. Age-related arterial changes—such as arterial stiffening and calcification—impair the body’s intrinsic blood pressure regulation mechanisms, leading to greater blood pressure variability and complicating HTN management [41].

Obesity was identified as a significant risk factor in the adjusted models for diastolic HTN, with an approximately nine-fold higher prevalence compared to individuals with underweight or normal weight. Wang et al. found a strong association between obesity and the prevalence of high blood pressure among adults aged ≥60 years in the United States, particularly when using central adiposity indicators such as the weight-adjusted waist index (WWI) [42]. This finding is consistent with pathophysiological mechanisms described in recent reviews, whereby visceral fat accumulation promotes systemic inflammation, insulin resistance, and endothelial dysfunction, contributing to increased arterial stiffness and elevated blood pressure. Similarly, Gao et al. reported a positive association between higher BMI and hypertension incidence in Latin America and sub-Saharan Africa, especially among older adults and socioeconomically vulnerable populations [43]. Evidence from longitudinal cohorts also indicates that excess adiposity in midlife predicts later hypertension onset and target-organ damage. These results underscore the need to integrate obesity screening and control into public health strategies for hypertension prevention, particularly in rapidly ageing and urbanising societies. Accordingly, all associations discussed in this study should be interpreted as descriptive and non-causal, given the cross-sectional nature of the analysis.

One of the main strengths of this study is the use of the DHS database, which, due to its probabilistic, stratified, and nationally representative design, allows for the generation of findings generalisable to the adult population in Peru. Moreover, given the standardised methodology of the DHS across countries, the associations observed in this study may be cautiously extrapolated to other low- and middle-income countries (LMICs) with similar demographic, nutritional, and epidemiological profiles. However, as this is a secondary data analysis, the study is limited in its ability to establish causal relationships and in its control of potential residual confounding. In this regard, important dietary confounders such as sodium intake, consumption of processed foods, caffeine intake, and adherence to overall dietary patterns (e.g., DASH) were not measured in the DHS and therefore could not be included in the models. Moreover, given the cross-sectional design of the study, the possibility of reverse causation cannot be ruled out and should be considered when interpreting the findings. Additionally, self-reported variables and dietary recall may be subject to misclassification and recall bias. In this regard, because dietary information on fruit and vegetable consumption was obtained through self-reported items from the DHS questionnaire and did not include information on portion sizes, exposure misclassification may have occurred; however, the operationalisation of intake was based on measures aligned with WHO recommendations. Furthermore, the analyses were conducted using a complete-case approach due to missing information on fruit and vegetable consumption and body mass index, which resulted in the exclusion of approximately 10% of the initially surveyed population. Although this level of missingness is comparable to that reported in other population-based dietary studies, the use of complete cases may have introduced selection bias, which cannot be entirely excluded despite the large analytical sample and transparent documentation of participant flow. In addition, information on antihypertensive medication use was not incorporated into the definition of hypertension, potentially leading to outcome misclassification and bias towards the null. Finally, key lifestyle factors such as physical activity, smoking, and alcohol consumption were not included, and despite the large sample size, the study may have had limited power to detect small effect sizes after multivariable adjustment.

## 5. Conclusions

In conclusion, fruit and vegetable consumption was associated with normal blood pressure in the unadjusted analysis; however, this association did not remain statistically significant after adjustment for potential confounders. These findings suggest that fruit and vegetable intake alone may not be independently associated with hypertension among the Peruvian population aged 15 years and older. In this context, future longitudinal studies considering broader dietary patterns are needed to better clarify the role of fruit and vegetable consumption in hypertension prevention.

## Figures and Tables

**Table 1:** General characteristics of respondents by blood pressure category in the Peruvian population, DHS 2022–2023 (*n* = 64,347).

	Normal	Pre-Hypertension	Hypertension	X^2^ (*p*-Value)
	*n* (%)	*n* (%)	*n* (%)	
**Overall**	33,189 (51.58%)	22,022 (34.22%)	9136 (14.20%)	
**Consumption of five servings of fruits and vegetables**				83.44 (<0.001)
<35	10,702 (48.92%)	7937 (36.29%)	3235 (14.79%)	
≥35	22,487 (52.95%)	14,085 (33.16%)	5901 (13.89%)	
**Sex**				3938.12 (<0.001)
Male	11,547 (37.76%)	13,556 (44.33%)	5479 (17.91%)	
Female	21,642 (64.10%)	8466 (25.07%)	3657 (10.83%)	
**Education level**				605.92 (<0.001)
No education	767 (41.12%)	603 (32.35%)	495 (26.54%)	
Primary	5010 (44.90%)	4037 (36.18%)	2110 (18.92%)	
Secondary	16,546 (54.66%)	9939 (32.83%)	3787 (12.51%)	
Higher	10,866 (51.61%)	7443 (35.35%)	2744 (13.03%)	
**Age**				7872.01 (<0.001)
15–19	5597 (76.6%)	1543 (21.11%)	167 (2.29%)	
20–29	8401 (64.58%)	3995 (30.71%)	612 (4.71%)	
30–39	7663 (57.99%)	4453 (33.70%)	1099 (8.31%)	
40–49	5295 (47.99%)	4152 (37.64%)	1586 (14.37%)	
50–59	3215 (37.06%)	3505 (40.40%)	1956 (22.54%)	
60+	3018 (27.17%)	4374 (39.38%)	3716 (33.45%)	
**Marital status**				1593.94 (<0.001)
Single	8591 (61.39%)	4279 (30.58%)	1123 (8.028%)	
Married	6726 (44.83%)	5495 (36.62%)	2784 (18.55%)	
Cohabiting	12,428 (53.63%)	8150 (35.17%)	2595 (11.20%)	
Widow/Divorced/Separated	5444 (44.71%)	4098 (33.66%)	2634 (21.63%)	
**Wealth Index**				485.07 (<0.001)
Poorest/Poorer	13,941 (56.34%)	8025 (32.43%)	2780 (11.23%)	
Middle	6978 (51.60%)	4600 (34.02%)	1945 (14.38%)	
Richer/Richest	12,270 (47.05%)	9397 (36.03%)	4411 (16.92%)	
**Region**				367.36 (<0.001)
Coastal	19,883 (49.02%)	14,192 (34.99%)	6488 (15.99%)	
Highlands	8592 (55.26%)	5210 (33.51%)	1747 (11.23%)	
Jungle	4714 (57.24%)	2619 (31.81%)	902 (10.95%)	
**Area of residence**				143.46 (<0.001)
Urban	26,587 (50.51%)	18,247 (34.66%)	7807 (14.83%)	
Rural	6602 (56.40%)	3775 (32.25%)	1329 (11.35%)	
**BMI**				8114.79 (<0.001)
Underweight/Normal	7088 (89.23%)	765 (9.62%)	91 (1.15%)	
Overweight	6722 (77.02%)	1679 (19.24%)	327 (3.74%)	
Obesity	19,379 (40.65%)	19,578 (41.07%)	8718 (18.29%)	

Variable names within the study are in bold.

**Table 2:** Factors associated with pre-hypertension and hypertension in the Peruvian population, DHS 2022–2023.

Variables	Pre-Hypertension						Hypertension					
	RRR	*p*-Value	95%CI	aRRR	*p*-Value	95%CI	RRR	*p*-Value	95%CI	aRRR	*p*-Value	95%CI
**Consumption of five servings of fruits and vegetables**												
<35	Ref.			Ref.			Ref.			Ref.		
≥35	0.84	<0.001	(0.79–0.89)	0.95	0.17	(0.89–1.02)	0.86	0.01	(0.79–0.94)	0.96	0.33	(0.87–1.05)
**Sex**												
Male	Ref.						Ref.			Ref.		
Female	0.33	<0.001	(0.31–0.35)	0.49	<0.001	(0.45–0.53)	0.35	<0.001	(0.32–0.39)	0.40	<0.001	(0.36–0.45)
**Education level**												
No Education	Ref.						Ref.			Ref.		
Primary	1.03	0.77	(0.86–1.22)	1.17	0.10	(0.97–1.41)	0.66	<0.001	(0.52–0.82)	0.90	0.40	(0.71–1.15)
Secondary	0.77	0.01	(0.65–0.90)	1.18	0.09	(0.97–1.43)	0.36	<0.001	(0.29–0.44)	0.96	0.72	(0.75–1.22)
Higher	0.87	0.12	(0.74–1.04)	1.16	0.15	(0.95–1.42)	0.39	<0.001	(0.32–0.49)	0.86	0.25	(0.66–1.11)
**Age**												
15–19	Ref.						Ref.			Ref.		
20–29	1.73	<0.001	(1.52–1.96)	2.00	<0.001	(1.71–2.33)	2.43	<0.001	(1.70–3.49)	3.30	<0.001	(2.27–4.81)
30–39	2.12	<0.001	(1.88–2.39)	2.60	<0.001	(2.21–3.04)	4.83	<0.001	(3.40–6.87)	7.46	<0.001	(5.11–10.89)
40–49	2.86	<0.001	(2.52–3.25)	3.56	<0.001	(3.01–4.20)	10.11	<0.001	(7.12–14.34)	15.74	<0.001	(10.77–23.00)
50–59	3.99	<0.001	(3.44–4.63)	3.79	<0.001	(3.14–4.55)	20.71	<0.001	(14.53–29.53)	23.35	<0.001	(15.84–34.41)
60+	5.32	<0.001	(4.65–6.08)	5.25	<0.001	(4.37–6.31)	42.05	<0.001	(29.65–59.63)	47.55	<0.001	(32.06–70.54)
**Marital status**												
Single	Ref.						Ref.			Ref.		
Married	1.64	<0.001	(1.50–1.80)	0.72	<0.001	(0.64–0.81)	3.17	<0.001	(2.74–3.68)	0.59	<0.001	(0.48–0.71)
Cohabiting	1.32	<0.001	(1.22–1.42)	0.82	<0.001	(0.74–0.92)	1.60	<0.001	(1.38–1.84)	0.65	<0.001	(0.54–0.76)
Widow/Divorced/Separated	1.52	<0.001	(1.38–1.67)	0.83	0.01	(0.74–0.95)	3.73	<0.001	(3.22–4.32)	0.80	0.02	(0.66–0.97)
**Wealth Index**												
Poorest/Poorer	Ref.						Ref.			Ref.		
Middle	1.15	<0.001	(1.06–1.24)	1.09	0.08	(0.99–1.19)	1.40	<0.001	(1.25–1.57)	1.26	0.01	(1.09–1.4)
Richer/Richest	1.33	<0.001	(1.25–1.42)	1.17	<0.001	(1.07–1.28)	1.81	<0.001	(1.65–1.99)	1.35	<0.001	(1.17–1.56)
**Region**												
Coastal	Ref.						Ref.			Ref.		
Highlands	0.85	<0.001	(0.80–0.90)	0.93	0.06	(0.87–1.00)	0.62	<0.001	(0.57–0.68)	0.70	<0.001	(0.63–0.79)
Jungle	0.78	<0.001	(0.73–0.83)	0.85	<0.001	(0.79–0.92)	0.59	<0.001	(0.53–0.65)	0.70	<0.001	(0.62–0.79)
**Area of residence**												
Urban	Ref.						Ref.			Ref.		
Rural	0.83	<0.001	(0.79–0.88)	0.93	0.06	(0.85–1.00)	0.68	<0.001	(0.62–0.75)	0.90	0.11	(0.80–1.02)
**BMI**												
Underweight/Normal	Ref.						Ref.			Ref.		
Overweight	2.31	<0.001	(2.01–2.66)	1.94	<0.001	(1.69–2.24)	3.78	<0.001	(2.56–5.58)	2.64	<0.001	(1.78–3.92)
Obesity	9.41	<0.001	(8.34–10.62)	4.22	<0.001	(3.68–4.85)	35.33	<0.001	(25.26–49.43)	7.16	<0.001	(5.04–10.19)

Variable names within the study are in bold.

**Table 3:** Factors associated with HTN subtypes in the Peruvian population, DHS 2022–2023.

	IDH						ISH						CSDH					
Variable	PR	*p*-Value	95%CI	aPR	*p*-Value	95%CI	PR	*p*-Value	95%CI	aPR	*p*-Value	95%CI	PR	*p*-Value	95%CI	aPR	*p*-Value	95%CI
**Consumption of five servings of fruits and vegetables**																		
<35	Ref.						Ref.			Ref.			Ref.			Ref.		
≥35	0.84	0.03	(0.72–0.98)	0.92	0.26	(0.78–1.07)	1.04	0.51	(0.92–1.18)	-	-	-	0.86	0.04	(0.77–0.99)	1.03	0.63	(0.92–1.13)
**Sex**																		
Male	Ref.						Ref.			Ref.			Ref.			Ref.		
Female	0.60	<0.001	(0.52–0.70)	0.79	0.02	(0.65–0.96)	0.77	<0.001	(0.68–0.87)	0.78	<0.001	(0.70–0.88)	0.44	<0.001	(0.38–0.50)	0.82	0.01	(0.73–0.92)
**Education level**																		
No Education	Ref.						Ref.			Ref.			Ref.			Ref.		
Primary	1.11	0.68	(0.68–1.80)	0.87	0.58	(0.53–1.4)	0.60	<0.001	(0.48–0.74)	0.85	0.11	(0.69–1.04)	0.93	0.59	(0.70–1.22)	-	-	-
Secondary	1.25	0.36	(0.78–1.98)	0.99	0.97	(0.60–1.63)	0.28	<0.001	(0.22–0.34)	0.75	0.01	(0.60–0.94)	0.79	0.09	(0.60–1.04)	-	-	-
Higher	1.74	0.02	(1.10–2.76)	1.12	0.65	(0.68–1.84)	0.26	<0.001	(0.20–0.32)	0.66	0.01	(0.51–0.84)	0.78	0.08	(0.59–1.03)	-	-	-
**Age**																		
15–19	Ref.						Ref.			Ref.			Ref.			Ref.		
20–29	2.14	0.01	(1.27–3.59)	2.20	0.01	(1.29–3.76)	1.69	0.04	(1.00–2.89)	1.93	0.02	(1.11–3.34)	2.73	0.07	(0.91–8.10)	1.82	0.03	(1.06–3.12)
30–39	4.15	<0.001	(2.52–6.82)	4.47	<0.001	(2.61–7.62)	1.66	0.05	(1.00–2.78)	1.96	0.02	(1.15–3.35)	7.30	<0.001	(2.45–21.77)	1.85	0.02	(1.09–3.14)
40–49	5.44	<0.001	(3.28–9.03)	5.76	<0.001	(3.32–9.98)	3.04	<0.001	(1.82–5.09)	3.29	<0.001	(1.90–5.70)	15.89	<0.001	(5.36–47.15)	3.17	<0.001	(1.84–5.46)
50–59	3.93	<0.001	(2.32–6.65)	3.64	<0.001	(2.07–6.40)	7.68	<0.001	(4.69–12.56)	6.18	<0.001	(3.64–10.50)	27.87	<0.001	(9.37–82.88)	6.06	<0.001	(3.60–10.21)
60+	1.83	0.04	(1.04–3.24)	1.68	0.12	(0.88–3.24)	22.85	<0.001	(14.21–36.75)	16.95	<0.001	(10.14–28.35)	21.95	<0.001	(7.40–65.08)	17.38	<0.001	(10.50–28.76)
**Marital status**																		
Single	Ref.						Ref.			Ref.			Ref.			Ref.		
Married	1.29	0.04	(1.01–1.66)	0.69	0.01	(0.52–0.92)	2.64	<0.001	(2.10–3.32)	0.75	0.02	(0.59–0.94)	2.78	<0.001	(2.22–3.50)	0.76	0.02	(0.60–0.95)
Cohabiting	1.32	0.02	(1.05–1.66)	0.75	0.03	(0.58–0.96)	1.13	0.32	(0.88–1.43)	0.71	0.01	(0.56–0.91)	1.78	<0.001	(1.42–2.26)	0.73	0.01	(0.57–0.93)
Widow/Divorced/Separated	1.29	0.06	(0.99–1.68)	0.94	0.71	(0.69–1.29)	3.89	<0.001	(3.09–4.88)	0.92	0.47	(0.73–1.16)	2.47	<0.001	(1.94–3.13)	0.95	0.64	(0.75–1.19)
**Wealth Index**																		
Poorest/Poorer	Ref.						Ref.			Ref.			Ref.			Ref.		
Middle	1.18	0.07	(0.99–1.42)	1.11	0.34	(0.90–1.36)	1.21	0.02	(1.04–1.42)	1.06	0.52	(0.89–1.26)	1.43	<0.001	(1.22–1.67)	1.01	0.92	(0.85–1.19)
Richer/Richest	1.38	<0.001	(1.16–1.63)	1.26	0.03	(1.02–1.55)	1.56	<0.001	(1.37–1.77)	1.11	0.22	(0.94–1.32)	1.53	<0.001	(1.34–1.74)	1.01	0.99	(0.85–1.17)
**Region**																		
Coastal	Ref.						Ref.			Ref.			Ref.			Ref.		
Highlands	1.26	0.01	(1.08–1.46)	1.52	<0.001	(1.30–1.80)	0.49	<0.001	(0.43–0.56)	0.49	<0.001	(0.43–0.57)	0.72	<0.001	(0.63–0.82)	0.49	<0.001	(0.43–0.57)
Jungle	0.70	<0.001	(0.58–0.8)	0.86	0.11	(0.71–1.03)	0.64	<0.001	(0.56–0.74)	0.73	<0.001	(0.63–0.84)	0.73	<0.001	(0.64–0.84)	0.72	<0.001	(0.63–0.83)
**Area of residence**																		
Urban	Ref.						Ref.			Ref.			Ref.			Ref.		
Rural	0.79	0.01	(0.68–0.92)	0.87	0.18	(0.72–1.06)	0.76	<0.001	(0.67–0.84)	1.02	0.80	(0.89–1.18)	0.76	<0.001	(0.67–0.86)	1.06	0.43	(0.92–1.22)
**BMI**																		
Underweight/Normal	Ref.						Ref.			Ref.			Ref.			Ref.		
Overweight	2.89	<0.001	(1.79–4.66)	2.20	0.01	(1.37–3.56)	2.67	0.06	(0.96–7.5)	2.30	0.11	(0.82–6.42)	5.65	<0.001	(2.74–11.68)	2.31	0.11	(0.83–6.46)
Obesity	4.24	<0.001	(2.79–6.43)	3.33	<0.001	(2.12–5.25)	38.06	<0.001	(16.16–89.63)	9.04	<0.001	(3.80–21.49)	40.23	<0.001	(21.78–74.32)	9.43	<0.001	(3.97–22.41)

Variable names within the study are in bold.

## Data Availability

The data used in this study are publicly available from the Demographic and Health Survey (DHS) through the INEI data portal (https://proyectos.inei.gob.pe/microdatos/ accessed on 31 November 2025), subject to standard registration and data use approval.
